# Associations of genetic variants in endocytic trafficking of epidermal growth factor receptor super pathway with risk of nonsyndromic cleft lip with or without cleft palate

**DOI:** 10.1002/mgg3.497

**Published:** 2018-11-08

**Authors:** Bing Li, Lan Ma, Chi Zhang, Zhixuan Zhou, Hua Yuan, Hongbing Jiang, Yongchu Pan, Qian Tan

**Affiliations:** ^1^ Department of Burns and Plastic Surgery, The Drum Tower Clinical Medical College Nanjing Medical University Nanjing China; ^2^ Department of Oral and Maxillofacial Surgery, Affiliated Hospital of Stomatology Nanjing Medical University Nanjing China; ^3^ Jiangsu Key Laboratory of Oral Diseases Nanjing Medical University Nanjing China; ^4^ Department of Orthodontics, Affiliated Hospital of Stomatology Nanjing Medical University Nanjing China; ^5^ Department of Polyclinic, Affiliated Hospital of Stomatology Nanjing Medical University Nanjing China; ^6^ State Key Laboratory of Reproductive Medicine Nanjing Medical University Nanjing China; ^7^ Department of Burns and Plastic Surgery, Affiliated Drum Tower Hospital Nanjing University Medical School Nanjing China

**Keywords:** endocytosis, epidermal growth factor receptor, genome‐wide association study, nonsyndromic cleft lip with or without cleft palate, pathway, single‐nucleotide polymorphism

## Abstract

**Background:**

The genetic etiology of nonsyndromic cleft lip with or without cleft palate (NSCL/P) has not been fully clarified to date. Epidermal growth factor receptor (EGFR) was reportedly involved in its biological establishment and regulation of cell migration during the embryonic stage.

**Methods:**

We selected a super pathway of endocytic trafficking of EGFR and investigated the associations of single‐nucleotide polymorphisms (SNPs) in the super pathway with the risk of NSCL/P by analyzing our published genome‐wide association study (GWAS) data from 504 NSCL/P individuals and 455 controls. After the false discovery rate (FDR) control, we conducted linkage disequilibrium (LD) analyses and conditional regression analyses to obtain independent lead SNPs. We performed LD analyses between the lead SNPs and the reported SNPs to find novel ones from our study. We annotated the lead SNPs and investigated their mapped genes in silico.

**Results:**

A total of 82 SNPs showed a statistical association with the risk of NSCL/P after FDR control. They contained three reported SNPs which were g.117068049G>A (rs7078160), g.117086783C>G (rs10886040), and g.117101266G>T (rs17095681). Four independent lead SNPs were obtained, including g.116979803 T>C (rs1905539) and g.117037960A>G (rs7902502) at 10q25.3, g.35720163G>C (rs75656820) at 17q12, and g.156864512G>A (rs1800877) at 1q23.1. Three of them were in low LD (*r* ^2^ < 0.5) with the reported SNPs except g.117037960A>G (rs7902502), so these three were newly identified. Lead SNPs were mapped to three genes: *SHTN1*,* AP2B1,* and *NTRK1*. The three genes were relatively more highly expressed in the human craniofacial region and in the proximal maxillary location during the craniofacial development stage of the embryonic mouse.

**Conclusion:**

Our results suggested that *SHTN1*,* AP2B1,* and *NTRK1* might be associated with the development of NSCL/P.

## 1 INTRODUCTION

Nonsyndromic cleft lip with or without cleft palate (NSCL/P) is a natal defect of orofacial malformation. Patients with NSCL/P may have an abnormal appearance of the lip, nose, palate, and dentition. They also have corresponding dysfunction and may have hearing problems or even psychological problems.

Nonsyndromic cleft lip with or without cleft palate is thought to be caused by genetic and environmental factors or their combined effects (Dixon, Marazita, Beaty, & Murray, 2011; Grosen et al., 2010; Sivertsen et al., 2008). Many studies were conducted to investigate the genetic variants associated with NSCL/P risk. Several large‐scale genome‐wide association studies (GWASs) or related meta‐analyses in different populations identified some underlying risk genes (Beaty et al., 2010; Birnbaum et al., 2009; Grant et al., 2009; Leslie et al., 2016; Leslie et al., 2017; Ludwig et al., 2012; Ludwig et al., 2017; Mangold et al., 2010; Sun et al., 2015; Wang et al., 2016; Yu et al., 2017).

Nevertheless, GWASs have basically been used to investigate the associations of single SNPs with phenotypes of interest, which to a certain degree neglect the gene function in the pathogenesis of the complex disease. As some diseases may result from combined action of multiple loci or multiple genes in a pathway or even respective pathways, a single SNP may cause only a tiny risk to the development of the disease. GWAS applied such a strict threshold (*p* < 5×10^‐8^) for statistical significance that more real but weaker associations that did not reach this standard exactly could be missed. So the pathway‐based or gene set enrichment analyses were introduced as a supplement to GWAS in recent years. The pathway‐based association analysis has been increasingly utilized as one of the effective complements to GWAS (Pan et al., 2017; Wang, Li, & Hakonarson, 2010; Weng et al., 2011; Zhong, Yang, Kaplan, Molony, & Schadt, 2010) by investigating genetic variants of some aggregate genes in given pathways (Ramanan, Shen, Moore, & Saykin, 2012; Uimari et al., 2017). This measure makes it possible to better understand the genetic variants and provides clues to biological processes in complex disease research. More risk contributors with weaker significance can be discovered.

Neural crest cells undergo a migration to the cranial region and differentiate into most of the craniofacial structural components such as bones, nerves, connective tissues, and dentins in the embryonic stage (Osumi‐Yamashita, Ninomiya, Doi, & Eto, 1994; Trainor and Tam, 1995). Epidermal growth factor receptor (EGFR) is one type of receptor tyrosine kinase (RTK). Its activated signal can trigger and mediate many biological events. It was reported that EGFR activation was required to establish the embryo axis (Queenan, Ghabrial, & Schupbach, 1997) and affect the fate of cells. It regulated cell migration in the embryonic developmental phase (Ciccolini, Mandl, Holzl‐Wenig, Kehlenbach, & Hellwig, 2005; Mellott & Burke, 2008), which is closely related to the development of craniofacial structure (Chai et al., 2000).

Previous studies in mouse suggested that EGFR might be involved in the development of cleft palate (Abbott et al., 2003; Miettinen et al., 1999). However, whether the genetic variants of the EGFR pathway are associated with the human NSCL/P has not been reported in the previous studies. The endocytic trafficking of the EGFR super pathway is one of the super pathways mediated by EGFR. This study used SNPs in the genes in this super pathway to investigate their associations with the risk of NSCL/P using our GWAS data.

## 2 MATERIALS AND METHODS

### 2.1 Ethical compliance

This study was approved by the institutional ethics committee of Nanjing Medical University (njmu2015–220).

### 2.2 Subjects

The subjects included 504 NSCL/P cases and 455 controls. They were all Han Chinese without kinships. All the physical examinations of NSCL/P individuals were performed by two qualified and experienced oral surgeons. Patients with orofacial clefts syndrome were excluded. Detailed medical records were made, and general information was documented, including age, gender, ethnicity, and family history. Informed written consents were signed by adult subjects themselves or guardians in the case of minors.

### 2.3 Genotyping, imputation, and quality controls

GWAS genotyping was performed using Affymetrix Axiom Genome‐Wide CHB1 & CHB2 Array Plates (by CapitalBio Corporation; Sun et al., 2015). Samples that did not meet the following criteria of standard quality control were excluded; (a) the samples had extreme autosomal heterozygosity rates (>6 *SD* from the mean), (b) the samples had call rates <95%, and (c) the genotyping results were not consistent with the gender records. The SNPs that did not meet the following criteria were excluded; (a) genotyping call rates were <95%, (b) minor allele frequency (MAF) was <0.05, (c) the Hardy–Weinberg Equilibrium (HWE) *P* value was <10^‐5^, and (d) not located at the autosomal loci. Imputation was performed by IMPUTE2 (https://mathgen.stats.ox.ac.uk/impute/impute_v2.html, imputation step) based on haplotype information from the 1,000 Genomes Project (https://www.1000genomes.org). Imputed SNPs were retained if quality info was >0.8.

### 2.4 Selection of genes and association analyses

Genes in the super pathway of endocytic trafficking of EGFR (Supporting Information Figure S1) were obtained based on GeneCards (https://www.genecards.org/). Autosomal genes were retained. Genetic variants of the selected genes were analyzed including 2‐kb upstream and downstream of genes based on GencodeV26lift37. Genotype data that passed through the quality control with imputation were used for the subsequent assessment. We employed PLINK 1.06 to detect associations of SNPs with the risk for NSCL/P, and we used the Benjamini‐Hochberg approach to control the false discovery rate (FDR) for multiple tests.

### 2.5 Linkage disequilibrium analyses and conditional regression analyses

Statistically significant SNPs (FDR *p* ≤ 0.05) were used to conduct linkage disequilibrium (LD) analyses using the 1,000 Genomes Project (https://www.1000genomes.org) and conditional regression analyses by PLINK 1.06. Several lead SNPs with independent signals were obtained. Each lead SNP had the lowest *p* value in significant SNPs with moderate to high LD (*r* ^2^ ≥ 0.5) with it, still maintaining significance in the conditional analysis when the other lead SNPs were treated as an index. We estimated LD between the lead SNPs and the reported SNPs that were published by GWAS of NSCL/P. The lead SNP was considered newly identified if it was in low or no LD with all the reported SNPs (*r* ^2 ^< 0.5; Lim, Ho, Chou, & Waye, 2011).

### 2.6 Annotation and prediction of the lead SNPs

We explored basic information about lead SNPs using two databases: dbSNP (https://www.ncbi.nlm.nih.gov/projects/SNP/) and UCSC Genome Browser (https://genome.ucsc.edu/). We predicted SNP function by two online tools: RegulomeDB (https://www.regulomedb.org/) and SNPinfo Web Server (https://snpinfo.niehs.nih.gov/). We investigated the lead SNP information in the integrative analysis of 127 reference human epigenomes based on uniformly processed datasets of ROADMAP epigenomics PROJECT (https://www.roadmapepigenomics.org/; Bernstein et al., 2010; Consortium 2012).

### 2.7 Gene expression in silico

The susceptibility SNPs were mapped to risk genes. We exploited the Genotype‐Tissue Expression (GTEx) project (https://www.gtexportal.org/home/; Consortium 2013) to query gene expression data (RNA‐seq) in 53 human tissues from 8,555 tissue samples of 570 postmortem human bodies. We also queried gene expression levels (microarray) in the proximal and distal location of maxilla and mandible of embryonic mouse based on GEO data (https://www.ncbi.nlm.nih.gov/geo/query/acc.cgi?acc=GSE67985) linked with FaceBase (https://www.facebase.org/).

### 2.8 Statistical analysis

General statistical analysis was carried out using PLINK 1.06 and R 3.4.1 (https://www.r-project.org/). Imputation was performed by IMPUTE2 (https://mathgen.stats.ox.ac.uk/impute/impute_v2.html, imputation step) based on the 1,000 Genomes Project (https://www.1000genomes.org). The associations between SNPs and the risk of NSCL/P were analyzed using an additive genetic model of PLINK 1.06. The odds ratios and 95% confidence intervals were estimated using an additive model in the logistic regression analyses. Benjamini‐Hochberg FDR procedure was employed to adjust *P* values. The Manhattan plot of *p* values of SNPs in different chromosomes was constructed relying on the qqman package in R3.4.1. LD analysis was performed by PLINK 1.06 based on the CHB&JPT of the 1,000 Genomes Project November 2014 release (https://www.1000genomes.org). Regional plots were made using Locuszoom (https://locuszoom.sph.umich.edu/).

The flowchart of this study is shown in Supporting Information Figure S2.

## 3 RESULTS

### 3.1 Association results

**Figure 1:**
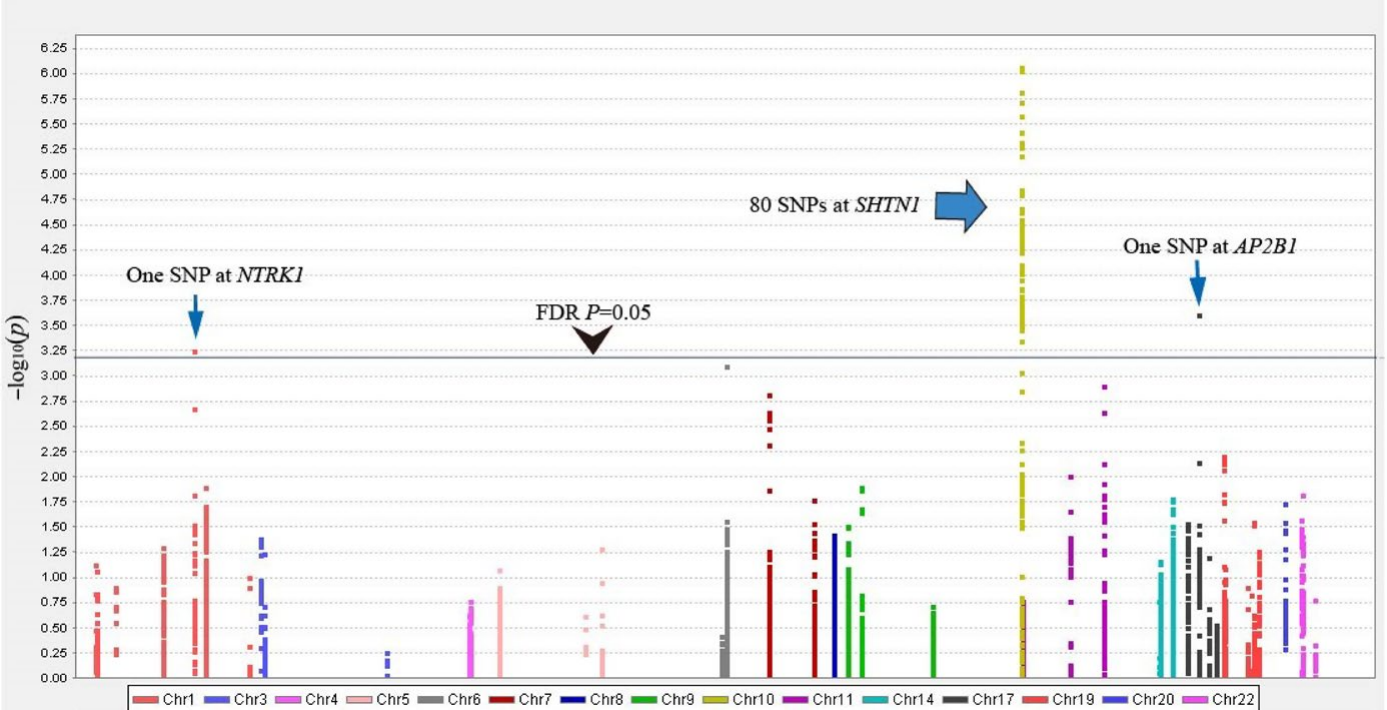
Manhattan plot of the associations of the SNPs of the super pathway genes with risk of nonsyndromic cleft lip with or without cleft palate. The *X*‐axis presented different chromosome loci, and the *Y*‐axis presented −log10 *p* values of SNPs. Eighty‐two SNPs attained a statistical association with the risk of NSCL/P after an adjustment for multiple testing (Benjamini–Hochberg FDR procedure, *p* ≤ 0.05)

The quality control was performed, and 4,844,517 SNPs were extracted from 504 cases and 455 controls including imputed data. Genes were selected from endocytic trafficking of EGFR super pathway (Supporting Information Table S1). Forty‐three genes remained after excluding five genes on the X chromosome. A total of 7,670 SNPs in 43 genes were evaluated. The Manhattan plot is shown in Figure 1. Eighty‐two SNPs mapping to three genes, *SHTN1* (OMIM 611171, NC_000010.11), *AP2B1* (OMIM 601025, NC_000017.11), and *NTRK1* (OMIM 191315, NC_000001.11; Supporting Information Table S2), showed a statistical association with the risk of NSCL/P after an adjustment for multiple testing (FDR *p* ≤ 0.05).

### 3.2 Identification of the independent lead SNPs

**Figure 2:**
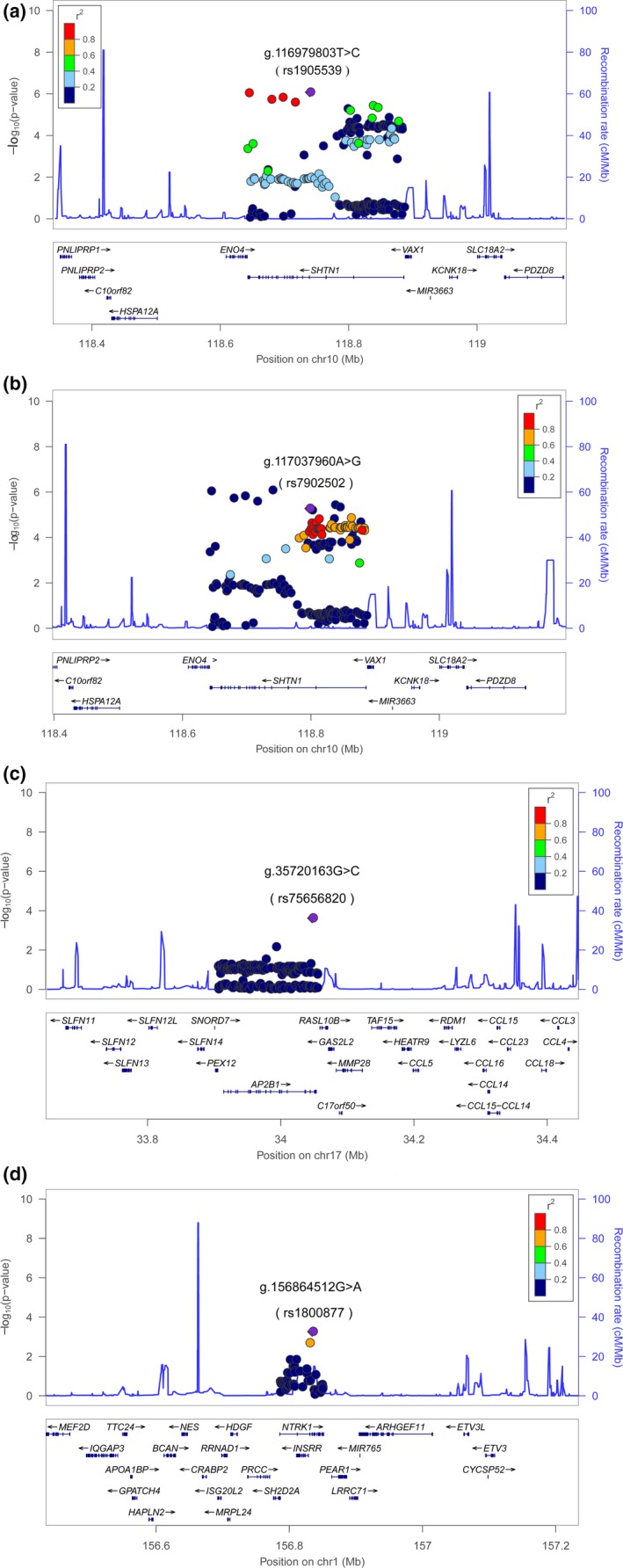
Regional plots of associations of three susceptibility loci with risk of NSCL/p. Figures showed association results of SNPs and recombination rates in three susceptibility loci (10q25.3, 17q12, and 1q23.1), and four lead SNPs were identified shown in a–d. For each plot, the −log10P values of the SNPs were presented according to the chromosomal positions of the SNPs. Estimated recombination rates were shown as light‐blue lines. The lead SNPs were presented in purple, and each of them was more significant than SNPs in moderate to high LD with it (*r* ^2^ ≥ 0.5). Other SNPs were colored according to their LD (*r* ^2^) with the lead SNP. The genes within or near the susceptibility region were annotated at the bottom, and their transcript direction was shown by arrows

Linkage disequilibrium analyses and conditional analyses were conducted for 82 statistically significant SNPs. Four lead SNPs were obtained: g.116979803T>C (rs1905539), g.117037960A>G (rs7902502), g.35720163G>C (rs75656820), and g.156864512G>A (rs1800877). They were in low LD (r^2^<0.5) with each other, and each one had the lowest *P* value in the moderate to high LD (*r* ^2^ ≥ 0.5) block among the 82 SNPs (Figure 2). No evidence of other independently significant signals was provided from the conditional regression analyses (Supporting Information Table S3). Lead SNP statistical associations with the risk of NSCL/P are presented in Table 1.

**Table 1:** Four lead SNPs showing statistical associations (FDR *p* ≤ 0.05) with the risk of nonsyndromic cleft lip with or without cleft palate

SNP	Chr Loci	OR (95% CI)	*p* d	*p*_FDR_ e	Gene
g.116979803T>C^a^ (rs1905539)	10q25.3	1.652 (1.353–2.018)	8.40E−07	3.544E−3	*SHTN1*
g.117037960A>G^a^ (rs7902502)	10q25.3	0.5125 (0.3843–0.6834)	5.30E−06	5.081E−3	*SHTN1*
g.35720163G>C^b^ (rs75656820)	17q12	0.4574 (0.3013–0.6944)	2.40E−04	2.334E−2	*AP2B1*
g.156864512G>A^c^ (rs1800877)	1q23.1	1.969 (1.340–2.892)	5.53E−04	4.823E−2	*NTRK1*

Note

CI: Confidence interval; Chr: Chromosome.

a NC_000010.11 (Homo sapiens chromosome 10, GRCh38.p12).

b NC_000017.11 (Homo sapiens chromosome 17, GRCh38.p12).

c NC_000001.11 (Homo sapiens chromosome 1, GRCh38.p12).

d Primary *p* value before multiple testing corrections.

e *p* Value after the false discovery rate (FDR) control.

Eight‐two significant SNPs included three that were reported by previous GWASs of NSCL/P, which were g.117101266G>T (rs17095681), g.117068049G>A (rs7078160), and g.117086783C>G (rs10886040). The lead SNP, g.117037960A>G (rs7902502), was in moderate to high LD with g.117101266G>T (rs17095681; *r* ^2^ = 0.72). The remaining three lead SNPs were in low LD with previously reported SNPs based on GWAS (*r* ^2^<0.5). These three SNPs: g.116979803T>C (rs1905539), g.35720163G>C (rs75656820), and g.156864512G>A (rs1800877) were considered newly identified.

### 3.3 Annotation and prediction of lead SNPs in silico

Among four lead SNPs, two (g.116979803T>C [rs1905539] and g.117037960A>G [rs7902502]) at 10q25.3 were mapped to *SHTN1*. One (g.35720163G>C [rs75656820]) at 17q12 was mapped to *AP2B1*. Another one (g.156864512G>A [rs1800877]) at 1q23.1 was mapped to *NTRK1* (Table 1). Lead SNPs were annotated and used to predict functions (Supporting Information Table S4). They were all mapped to introns.

**Figure 3:**
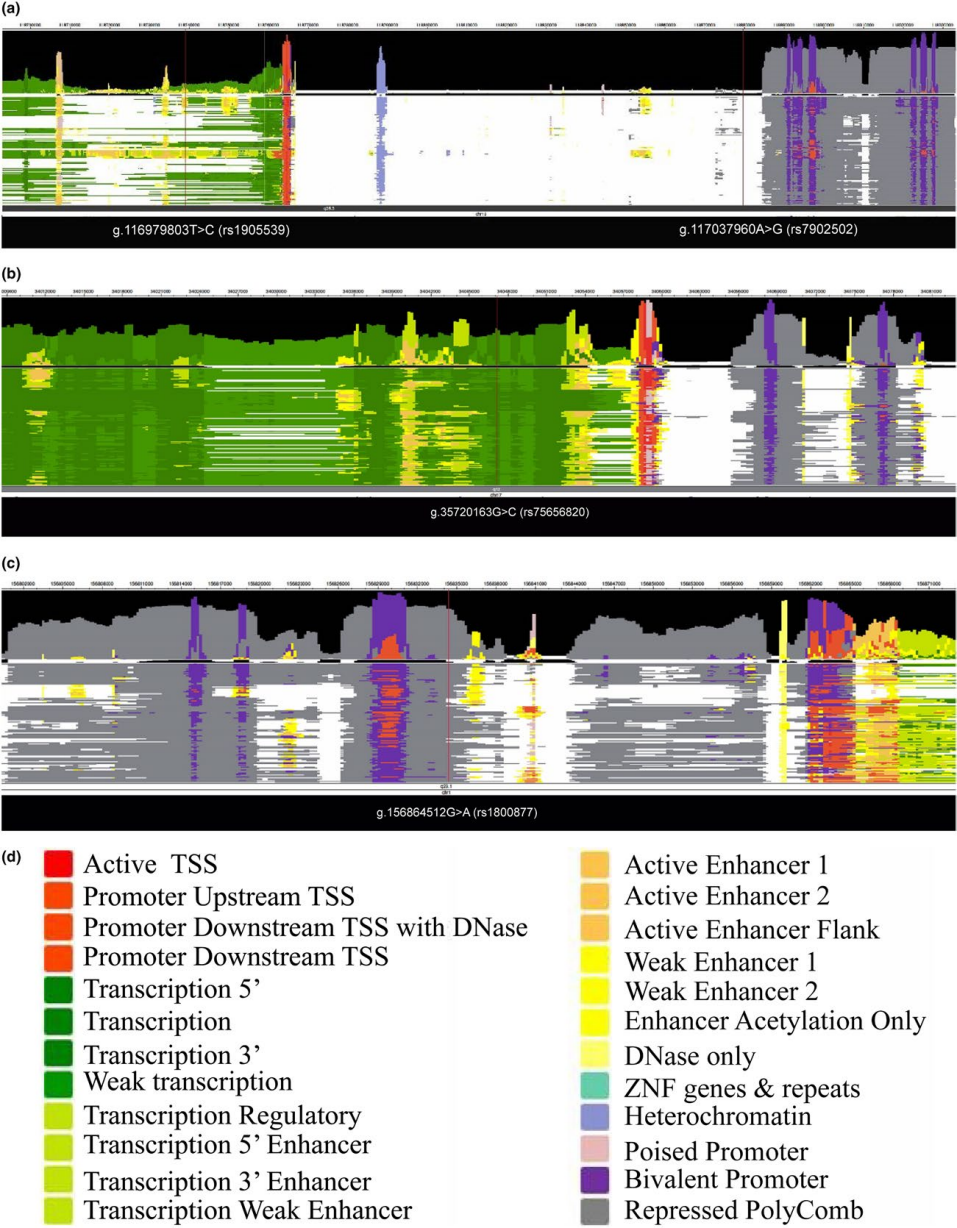
Information of four lead SNPs in 127 reference human epigenomes. (a) Two lead SNPs at 10q25.3 (b) One lead SNP at17q12 (c) one lead SNP at 1q23.1. Four lead SNPs were presented in 127 consolidated human epigenomes based on uniformly processed data sets of Roadmap Epigenomics Project. Annotation for configuring colors was listed in the bottom (d)

We obtained information on four lead SNPs in 127 consolidated human epigenomes using the Roadmap epigenomics Project (Figure 3). The SNP g.117037960A>G (rs7902502) of *SHTN1* did not map to a functional region of the chromosome. Three newly identified lead SNPs were presented as follows: g.116979803T>C (rs1905539) of *SHTN1* mapped to the transcription, transcription regulatory regions, or transcription enhancers in some of epigenomes. g.35720163G>C (rs75656820) of *AP2B1* mostly mapped to transcription regions. g.156864512G>A (rs1800877) of *NTRK1* mapped to elements repressed by polycomb in the reference epigenomes.

### 3.4 Expression of the mapped genes

Expression of the associated genes, *SHTN1*,* AP2B1,* and *NTRK1*, in 53 different tissues from the GTEx data of 8,555 samples (570 subjects) is shown in Supporting Information Figure S3. Both *SHTN1* and *AP2B1* had the highest median expression levels in brain tissues. *NTRK1* had relatively high median expression levels in the urinogenital and brain tissues.

**Figure 4:**
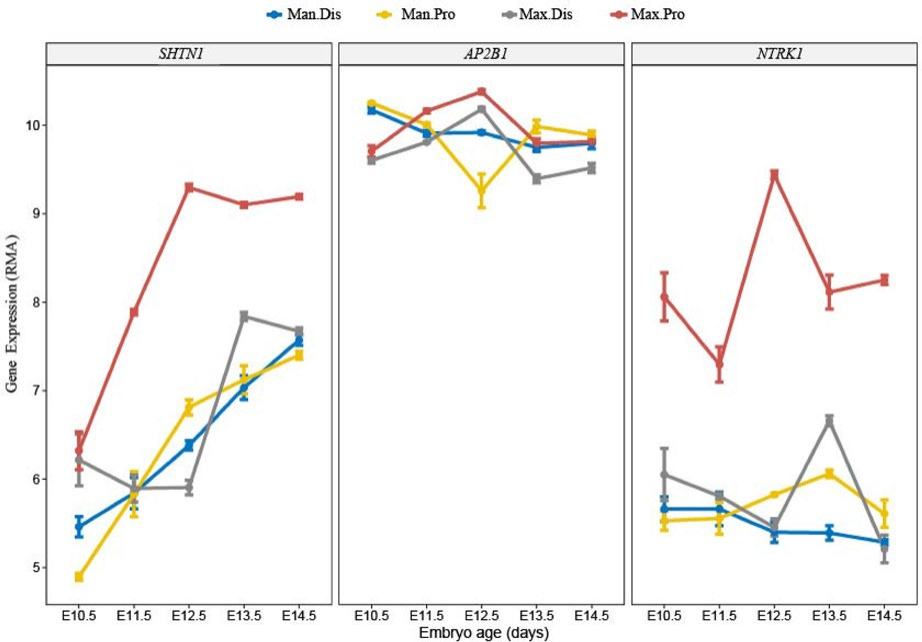
Three genes expressions in proximal and distal positions of the maxilla and mandible in mouse embryonic stage. Man.Dis: Mandibular distal location; Man.Pro: Mandibular proximal location; Max.Dis: Maxillary distal location; Max.Pro: Maxillary proximal location. Figures showed median levels of *SHTN1, AP2B1,* and *NTRK1* expressions in proximal and distal locations of maxilla and mandible during the 10.5th day to 14.5th day of mouse embryonic stage based on Geo data (GSE67985)

The expression of three genes in the proximal and distal locations of the maxilla and mandible during the mouse embryonic stage based on the data from GEO is presented in Figure 4. *SHTN1* and *NTRK1* kept the highest median expression levels in the proximal maxillary location during the observation period of embryonic 10.5th to 14.5th day. *AP2B1* had the highest median levels in the proximal position of the maxilla compared with the other positions on the 11.5th and 12.5th day of the embryonic stage.

## 4 DISCUSSION

Epidermal growth factor receptor is one member of the ErbB family, which is an RTK. Activated EGFR is involved in proliferation, differentiation, and cell fate determination. When RTK is bound by the ligand, it activates itself through autophosphorylation, and the receptor is internalized. It stimulates downstream signals such as Ras/MAPK cascades to promote different biological events (Goh & Sorkin, 2013; Goh, Huang, Kim, Gygi, & Sorkin, 2010).

In this study, 7,670 SNPs of genes in the super pathway were analyzed. Eighty‐two SNPs showed a statistical association with NSCL/P. Four lead SNPs were obtained, three of which were newly identified. These four lead SNPs mapped to introns were likely to bind to transcription factors (Supporting Information Table S4). Three newly identified SNPs (g.116979803T>C [rs1905539], g.35720163G>C [rs75656820], and g.156864512G>A [rs1800877]) could be in the transcription region or affect (enhance or repress) transcription in the reference human epigenomes (Figure 3). Introns are removed by RNA splicing either after or concurrent with transcription (Tilgner et al., 2012)*.* Introns can be transcribed into precursor messenger RNA (pre‐mRNA). Finally, they are removed, and exons are joined together; thereby the mature messenger RNA (mRNA) was made. Some introns on DNA regulate gene transcription by binding to transcription factors and hence function to control the expression of genes. These DNA sequences of introns can be enhancers, promoters, or silencers (Maston, Evans, & Green, 2006). The intronic splicing enhancers or silencers on the pre‐mRNA transcripts can also regulate RNA splicing by binding to the corresponding proteins (repressors and activators). The alternative splicing has an effect on increasing or reducing the probability that a nearby site will be used as a splice junction (Black, 2003). Therefore the significant intronic variants have the likelihood of affecting the risk genes expressions.

In the current study, two of the lead SNPs were mapped to *SHTN1*. There were three SNPs at *SHTN1* reported by previous GWASs: g.117068049G>A (rs7078160; Ludwig et al., 2012; Mangold et al., 2010; Sun et al., 2015), g.117086783C>G (rs10886040; Leslie et al., 2017), and g.117101266G>T (rs17095681; Wang et al., 2016). Most researchers believed that the risk gene was the adjacent gene *VAX1*, not *SHTN1*, because the SNP of *SHTN1* is located downstream of VAX1 and the homozygous mutations of *VAX1* led to mouse craniofacial malformations, including a cleft palate (Hallonet, Hollemann, Pieler, & Gruss, 1999). In our study, 80 SNPs at *SHTN1,* including three reported SNPs, showed statistical significance after multiple testing corrections. The newly identified lead SNP with the highest significance, g.116979803T>C (rs1905539), was mapped to an intron of *SHTN1*. Another significant SNP, g.116884159G>A (rs2257791) in strong LD (*r* ^2^ = 0.92) with lead SNP g.116979803T>C (rs1905539), had nearly the same *P* value as that of g.116979803T>C (rs1905539) (Figure 2a, Supporting Information Table S2). The SNP g.116884159G>A (rs2257791) was located in 3’‐UTR of another gene, *ENO4*, which may be required for sperm motility and function. Here, we would prefer *SHTN1* to be a susceptibility gene rather than *VAX1*.

*SHTN1* is involved in the super pathway of endocytic trafficking of EGFR (Supporting Information Figure S1) by participating in the recycling of *L1CAM*, which has a strong involvement in cell migration and nervous system development. SHTN1 protein functions on regulating the migration of neurons, forming a protrusion that attaches to the substrate, such as the extracellular matrix, so that the cells can move orderly from one site to another. SHTN1 plays a role in establishing neuronal polarization and contributes to axonal genesis, outgrowth, and morphogenesis. It mediates netrin‐1 (NTN1)‐induced F‐actin‐substrate coupling or clutch engagement within the axon growth cone (Liu et al., 2004; Ren et al., 2004), converting the F‐actin retrograde flow into traction forces, concomitantly with filopodium extension and axon outgrowth. *NTN1* was also reported as a susceptibility gene of NSCL/P in three previous GWASs (Leslie et al., 2017; Sun et al., 2015; Yu et al., 2017).

The lead SNP, g.156864512G>A (rs1800877), located in 1q23.1, was mapped to* NTRK1*, which has not been previously reported to be susceptible. *NTRK1* encodes neurotrophic receptor tyrosine kinase 1, which is an RTK that has a high affinity for NGF. When NTRK1 is bound by the ligand, it is internalized via endocytosis. It phosphorylates itself and promotes downstream signaling so it can support axonal extension. It can promote neuron survival and cell differentiation in the developing nervous system. An absence of ligand activation may promote cell death. Mutations in this gene were associated with insensitivity to pain, cognitive disability, and anhidrosis.

Another lead SNP, g.35720163G>C (rs75656820), located in 17q12, was mapped to *AP2B1*. The protein encoded by this gene is one of two large chain components of the adaptor protein complex 2 (AP‐2), which serves to link clathrin to receptors in coated vesicles. This gene is indispensable in receptor‐mediated endocytosis (clathrin‐dependent endocytosis). *AP2B1* was never identified by previous NSCL/P GWASs. It was known that one syndrome (Char Syndrome) associated with this gene can be shown by facial malformations involving abnormal looks on the upper lip and nose: a shortened distance between the nose and upper lip, a triangular‐shaped mouth, and a flat and broad nose tip. These characteristics share some similarities with NSCL/P characteristics.

The three genes above were thought to be risk genes based on our study. These three genes aggregate in a super pathway regulating the endocytosis of EGFR, which is one of the RTKs. The common biological process of the three risk genes is nervous system development mediated by RTK endocytosis. We noted several other genes that were published by previous GWASs and have some connections with our results. For example, *SPRY2* (Leslie et al., 2017; Ludwig et al., 2012; Yu et al., 2017) encodes a protein that has a bimodal regulatory effect on EGFR/MAPK signaling. *EPHA3* (Ludwig et al., 2012) encodes a protein that belongs to the largest known subfamily of RTK, which particularly serves to mediate developmental events in the nervous system.

To discuss gene expression, we described briefly here how the cleft lip forms during development. In the early human embryonic stage, neural crest cells migrate into the pharyngeal arch. Later, the first pharyngeal arch develops to craniofacial structures. The medial nasal prominences emerge at the end of the 4th week, and they grow rapidly in the 5th week of the embryo. At the 6th to 7th week, the medial nasal prominences fuse with the maxillary prominences and form the upper lip. The medial nasal prominences develop into the philtrum of the lip, four incisor teeth, and the primary palate. The maxillary prominence develops to the rest of upper lip, maxillary bone, and soft tissues. A cleft lip occurs if the medial nasal prominences fail to fuse correctly with the proximal part of maxillary prominences. The 10.5th day of the mouse embryonic stage is analogous to the 28th day of human development, and the 13th to 14th day of mouse development is analogous to the 6th to 7th week of human development (https://embryology.med.unsw.edu.au/embryology/index.php/Mouse_Timeline_Detailed). From our study, the expressions of the three genes at risk in the mouse embryo all showed high median expression in the proximal position of maxilla on the 12.5th day of the embryonic stage (Figure 4). This outcome occurred just before the formation of the upper lip, which coincided with the pathological region of the NSCL/P occurrence.

Additionally, the gene expression of different human tissues (Supporting Information Figure S3) presenting the highest median expression of *SHTN1* and *AP2B1* were both in the brain. Most brain tissues had higher median expression levels of *SHTN1* and *AP2B1*. Although the highest median level of *NTRK1* was not in the cephalic tissue, some brain tissue had relative higher median expression of *NTRK1*. This outcome suggests that the three candidate genes in our study might influence NSCL/P risk by participating in craniofacial nervous system development.

In summary, this study may provide a biological and pathogenic cue for NSCL/P. The genetic variants of the three genes in the investigated super pathway showed statistical evidence for association with NSCL/P risk. These three genes were *SHTN1*,* AP2B1,* and *NTRK1*. Taken together, they suggest that the neurite‐oriented outgrowth and neuron migration or survival mediated by RTKs endocytosis signaling in the nervous system development of craniofacial structures might have an association with the risk of NSCL/P. Further studies are needed to elucidate the underlying mechanisms or detailed biological associations for this speculation.

## CONFLICTS OF INTEREST

None to declare.
