# Supplementary figures and images for: Associations of genetic variants in endocytic trafficking of epidermal growth factor receptor super pathway with risk of nonsyndromic cleft lip with or without cleft palate
Source: Mol Genet Genomic Med. 2018 Nov 8;6(6):1157–67. doi: 10.1002/mgg3.497 (PMC6305670; doi:10.1002/mgg3.497)

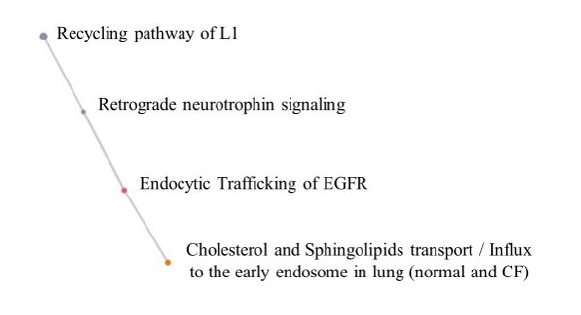

Supplement: Supplementary file 1 [file MGG3-6-1157-s001.tif]

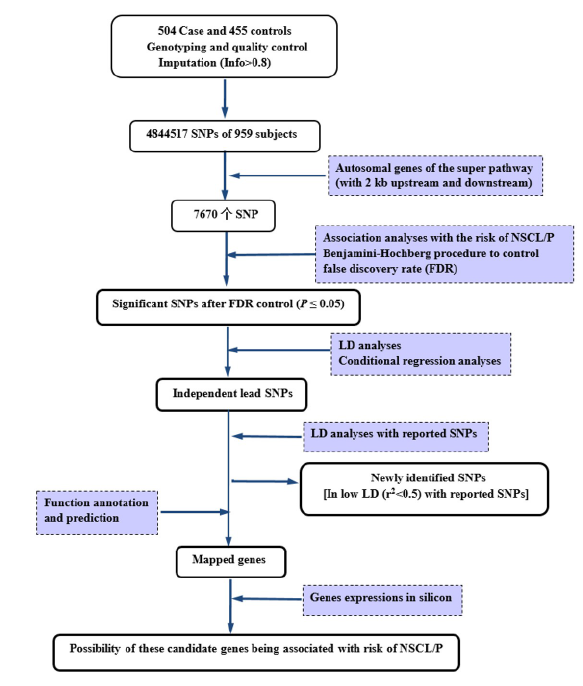

Supplement: Supplementary file 2 [file MGG3-6-1157-s002.tif]

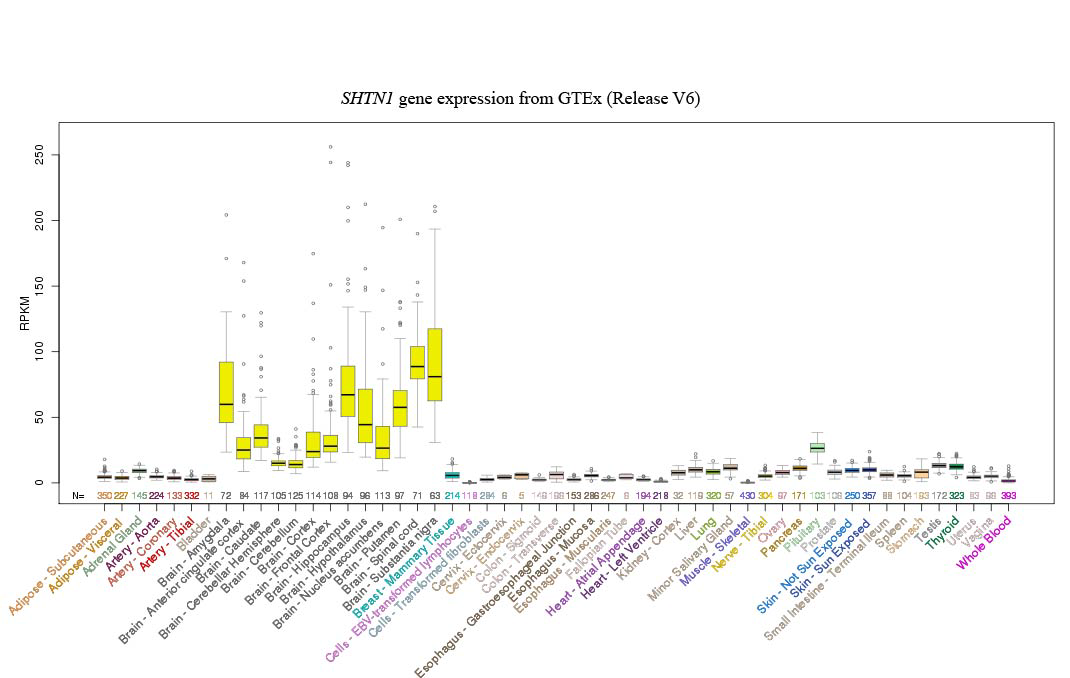

Supplement: Supplementary file 3 [file MGG3-6-1157-s003.tif]

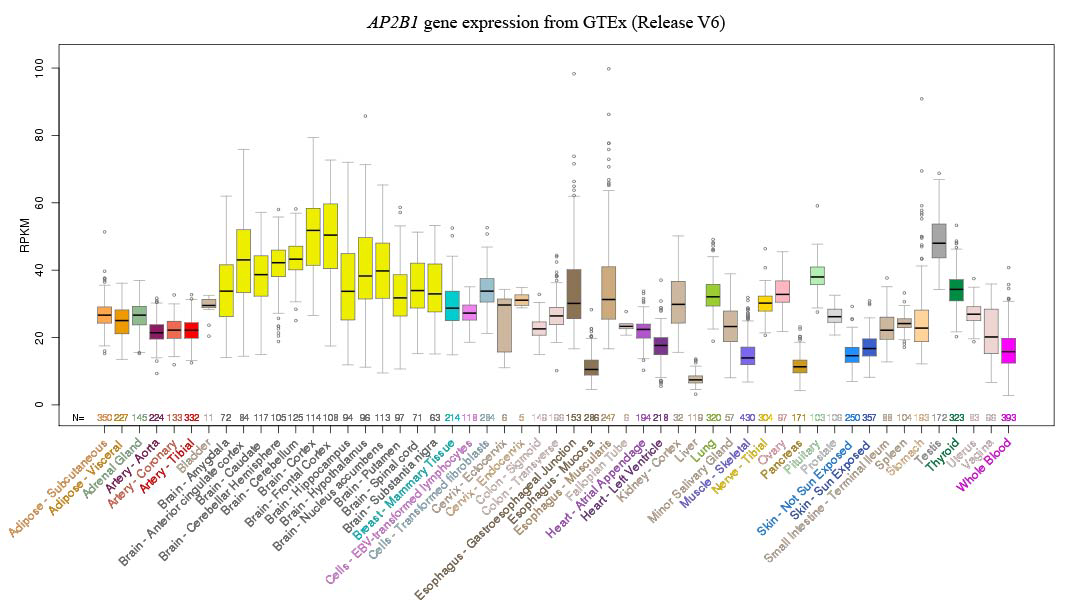

Supplement: Supplementary file 4 [file MGG3-6-1157-s004.tif]

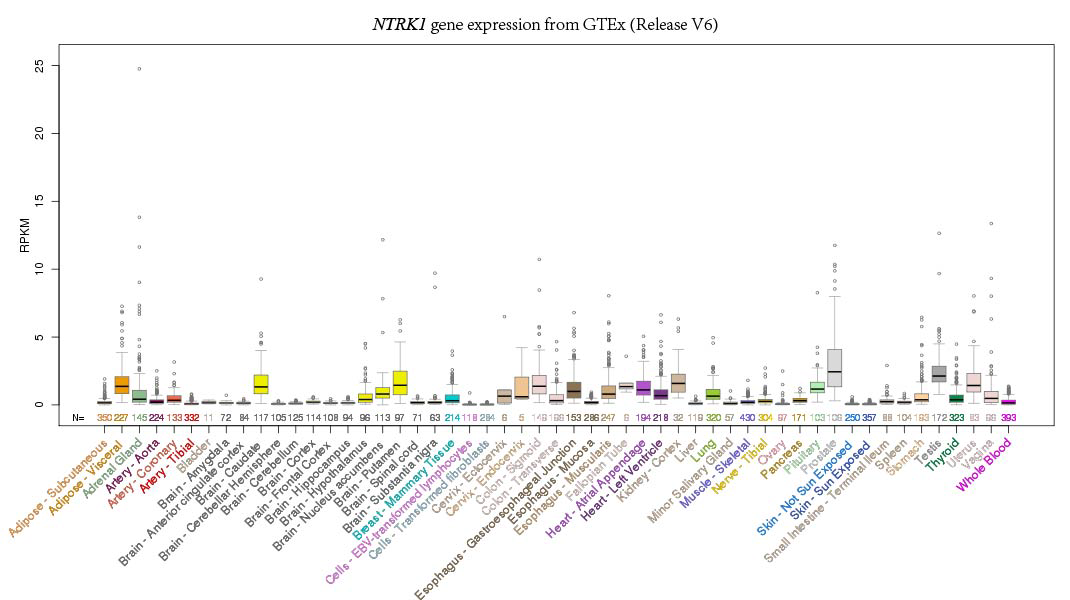

Supplement: Supplementary file 5 [file MGG3-6-1157-s005.tif]
