# Supplementary material for: Associations of genetic variants in endocytic trafficking of epidermal growth factor receptor super pathway with risk of nonsyndromic cleft lip with or without cleft palate
Source: Mol Genet Genomic Med. 2018 Nov 8;6(6):1157–67. doi: 10.1002/mgg3.497 (PMC6305670; doi:10.1002/mgg3.497)
Supplement: Supplementary file 6 [file MGG3-6-1157-s006.doc]

**Supplementary Table 1. 48 genes in the endocytic trafficking of EGFR super pathway**

| **Gene** | **Chr: Position^#^** |
| --- | --- |
| [*AP2A1*](http://genecards.org/cgi-bin/carddisp.pl?gene=AP2A1) | [19:49766968-49807113](http://mar2017.archive.ensembl.org/Homo_sapiens/Location/View?r=19:49766968-49807113:1) |
| [*CLTB*](http://genecards.org/cgi-bin/carddisp.pl?gene=CLTB) | [5:176392455-176416569](http://mar2017.archive.ensembl.org/Homo_sapiens/Location/View?r=5:176392455-176416569:-1) |
| [*RPS6KA5*](http://genecards.org/cgi-bin/carddisp.pl?gene=RPS6KA5) | [14:90847862-91060636](http://mar2017.archive.ensembl.org/Homo_sapiens/Location/View?r=14:90847862-91060636:-1) |
| [*LDLRAP1*](http://genecards.org/cgi-bin/carddisp.pl?gene=LDLRAP1) | [1:25543580-25568886](http://mar2017.archive.ensembl.org/Homo_sapiens/Location/View?r=1:25543580-25568886:1) |
| [*AP2A2*](http://genecards.org/cgi-bin/carddisp.pl?gene=AP2A2) | [11:924894-1012245](http://mar2017.archive.ensembl.org/Homo_sapiens/Location/View?r=11:924894-1012245:1) |
| [*DPYSL2*](http://genecards.org/cgi-bin/carddisp.pl?gene=DPYSL2) | [8:26514022-26658178](http://mar2017.archive.ensembl.org/Homo_sapiens/Location/View?r=8:26514022-26658178:1) |
| [*KIF4A*](http://genecards.org/cgi-bin/carddisp.pl?gene=KIF4A)*** | [X:70290090-70420832](http://mar2017.archive.ensembl.org/Homo_sapiens/Location/View?r=X:70290090-70420832:1) |
| [*RAB4B*](http://genecards.org/cgi-bin/carddisp.pl?gene=RAB4B) | [19:40778216-40796938](http://mar2017.archive.ensembl.org/Homo_sapiens/Location/View?r=19:40778216-40796938:1) |
| [*AP2B1*](http://genecards.org/cgi-bin/carddisp.pl?gene=AP2B1) | [17:35578046-35726409](http://mar2017.archive.ensembl.org/Homo_sapiens/Location/View?r=17:35578046-35726409:1) |
| [*L1CAM*](http://genecards.org/cgi-bin/carddisp.pl?gene=L1CAM)*** | [X:153861514-153909223](http://mar2017.archive.ensembl.org/Homo_sapiens/Location/View?r=X:153861514-153909223:-1) |
| [*RPS6KA6*](http://genecards.org/cgi-bin/carddisp.pl?gene=RPS6KA6)*** | [X:84058346-84187907](http://mar2017.archive.ensembl.org/Homo_sapiens/Location/View?r=X:84058346-84187907:-1) |
| [*RBSN*](http://genecards.org/cgi-bin/carddisp.pl?gene=RBSN) | [3:15070073-15099163](http://mar2017.archive.ensembl.org/Homo_sapiens/Location/View?r=3:15070073-15099163:-1) |
| [*AP2M1*](http://genecards.org/cgi-bin/carddisp.pl?gene=AP2M1) | [3:184174689-184184091](http://mar2017.archive.ensembl.org/Homo_sapiens/Location/View?r=3:184174689-184184091:1) |
| [*MSN*](http://genecards.org/cgi-bin/carddisp.pl?gene=MSN)*** | [X:65588377-65741931](http://mar2017.archive.ensembl.org/Homo_sapiens/Location/View?r=X:65588377-65741931:1) |
| [*SHTN1*](http://genecards.org/cgi-bin/carddisp.pl?gene=SHTN1) | [10:116881482-117126586](http://mar2017.archive.ensembl.org/Homo_sapiens/Location/View?r=10:116881482-117126586:-1) |
| [*EGF*](http://genecards.org/cgi-bin/carddisp.pl?gene=EGF) | [4:109912884-110012266](http://mar2017.archive.ensembl.org/Homo_sapiens/Location/View?r=4:109912884-110012266:1) |
| [*AP2S1*](http://genecards.org/cgi-bin/carddisp.pl?gene=AP2S1) | [19:46838136-46850992](http://mar2017.archive.ensembl.org/Homo_sapiens/Location/View?r=19:46838136-46850992:-1) |
| [*MAPK1*](http://genecards.org/cgi-bin/carddisp.pl?gene=MAPK1) | [22:21754500-21867680](http://mar2017.archive.ensembl.org/Homo_sapiens/Location/View?r=22:21754500-21867680:-1) |
| [*KIF4B*](http://genecards.org/cgi-bin/carddisp.pl?gene=KIF4B) | [5:155013755-155018132](http://mar2017.archive.ensembl.org/Homo_sapiens/Location/View?r=5:155013755-155018132:1) |
| [*EGFR*](http://genecards.org/cgi-bin/carddisp.pl?gene=EGFR) | [7:55019021-55211628](http://mar2017.archive.ensembl.org/Homo_sapiens/Location/View?r=7:55019021-55211628:1) |
| [*CLTA*](http://genecards.org/cgi-bin/carddisp.pl?gene=CLTA) | [9:36190856-36304781](http://mar2017.archive.ensembl.org/Homo_sapiens/Location/View?r=9:36190856-36304781:1) |
| [*RDX*](http://genecards.org/cgi-bin/carddisp.pl?gene=RDX) | [11:110174880-110296722](http://mar2017.archive.ensembl.org/Homo_sapiens/Location/View?r=11:110174880-110296722:-1) |
| [*LOC101928143*](http://genecards.org/cgi-bin/carddisp.pl?gene=LOC101928143) | [14:73460935-73463642](http://mar2017.archive.ensembl.org/Homo_sapiens/Location/View?r=14:73460935-73463642:-1) |
| [*EPN1*](http://genecards.org/cgi-bin/carddisp.pl?gene=EPN1) | [19:55675226-55709858](http://mar2017.archive.ensembl.org/Homo_sapiens/Location/View?r=19:55675226-55709858:1) |
| [*CLTC*](http://genecards.org/cgi-bin/carddisp.pl?gene=CLTC) | [17:59619689-59696956](http://mar2017.archive.ensembl.org/Homo_sapiens/Location/View?r=17:59619689-59696956:1) |
| [*RPS6KA1*](http://genecards.org/cgi-bin/carddisp.pl?gene=RPS6KA1) | [1:26529761-26575030](http://mar2017.archive.ensembl.org/Homo_sapiens/Location/View?r=1:26529761-26575030:1) |
| [*CAV1*](http://genecards.org/cgi-bin/carddisp.pl?gene=CAV1) | [7:116524785-116561184](http://mar2017.archive.ensembl.org/Homo_sapiens/Location/View?r=7:116524785-116561184:1) |
| [*EPN2*](http://genecards.org/cgi-bin/carddisp.pl?gene=EPN2) | [17:19215615-19336715](http://mar2017.archive.ensembl.org/Homo_sapiens/Location/View?r=17:19215615-19336715:1) |
| [*DNM2*](http://genecards.org/cgi-bin/carddisp.pl?gene=DNM2) | [19:10718079-10833488](http://mar2017.archive.ensembl.org/Homo_sapiens/Location/View?r=19:10718079-10833488:1) |
| [*RPS6KA2*](http://genecards.org/cgi-bin/carddisp.pl?gene=RPS6KA2) | [6:166409364-166906451](http://mar2017.archive.ensembl.org/Homo_sapiens/Location/View?r=6:166409364-166906451:-1) |
| [*CAV2*](http://genecards.org/cgi-bin/carddisp.pl?gene=CAV2) | [7:116287380-116508541](http://mar2017.archive.ensembl.org/Homo_sapiens/Location/View?r=7:116287380-116508541:1) |
| [*EPN3*](http://genecards.org/cgi-bin/carddisp.pl?gene=EPN3) | [17:50532543-50543750](http://mar2017.archive.ensembl.org/Homo_sapiens/Location/View?r=17:50532543-50543750:1) |
| [*DNM1*](http://genecards.org/cgi-bin/carddisp.pl?gene=DNM1) | [9:128191655-128255248](http://mar2017.archive.ensembl.org/Homo_sapiens/Location/View?r=9:128191655-128255248:1) |
| [*RPS6KA3*](http://genecards.org/cgi-bin/carddisp.pl?gene=RPS6KA3)*** | [X:20149911-20267100](http://mar2017.archive.ensembl.org/Homo_sapiens/Location/View?r=X:20149911-20267100:-1) |
| [*DAB2*](http://genecards.org/cgi-bin/carddisp.pl?gene=DAB2) | [5:39371675-39462300](http://mar2017.archive.ensembl.org/Homo_sapiens/Location/View?r=5:39371675-39462300:-1) |
| [*EPS15*](http://genecards.org/cgi-bin/carddisp.pl?gene=EPS15) | [1:51354263-51519328](http://mar2017.archive.ensembl.org/Homo_sapiens/Location/View?r=1:51354263-51519328:-1) |
| [*SH3GL2*](http://genecards.org/cgi-bin/carddisp.pl?gene=SH3GL2) | [9:17579082-17797129](http://mar2017.archive.ensembl.org/Homo_sapiens/Location/View?r=9:17579082-17797129:1) |
| [*EZR*](http://genecards.org/cgi-bin/carddisp.pl?gene=EZR) | [6:158765741-158819412](http://mar2017.archive.ensembl.org/Homo_sapiens/Location/View?r=6:158765741-158819412:-1) |
| [*LDLR*](http://genecards.org/cgi-bin/carddisp.pl?gene=LDLR) | [19:11089362-11133816](http://mar2017.archive.ensembl.org/Homo_sapiens/Location/View?r=19:11089362-11133816:1) |
| [*NGF*](http://genecards.org/cgi-bin/carddisp.pl?gene=NGF) | [1:115285918-115338236](http://mar2017.archive.ensembl.org/Homo_sapiens/Location/View?r=1:115285918-115338236:-1) |
| [*SRC*](http://genecards.org/cgi-bin/carddisp.pl?gene=SRC) | [20:37344685-37406050](http://mar2017.archive.ensembl.org/Homo_sapiens/Location/View?r=20:37344685-37406050:1) |
| [*NUMB*](http://genecards.org/cgi-bin/carddisp.pl?gene=NUMB) | [14:73275107-73463642](http://mar2017.archive.ensembl.org/Homo_sapiens/Location/View?r=14:73275107-73463642:-1) |
| [*RAB4A*](http://genecards.org/cgi-bin/carddisp.pl?gene=RAB4A) | [1:229271062-229305894](http://mar2017.archive.ensembl.org/Homo_sapiens/Location/View?r=1:229271062-229305894:1) |
| [*NTRK1*](http://genecards.org/cgi-bin/carddisp.pl?gene=NTRK1) | [1:156815640-156881850](http://mar2017.archive.ensembl.org/Homo_sapiens/Location/View?r=1:156815640-156881850:1) |
| [*DNM3*](http://genecards.org/cgi-bin/carddisp.pl?gene=DNM3) | [1:171841498-172418466](http://mar2017.archive.ensembl.org/Homo_sapiens/Location/View?r=1:171841498-172418466:1) |
| [*RPS6KA4*](http://genecards.org/cgi-bin/carddisp.pl?gene=RPS6KA4) | [11:64359148-64372215](http://mar2017.archive.ensembl.org/Homo_sapiens/Location/View?r=11:64359148-64372215:1) |
| [*RAB5A*](http://genecards.org/cgi-bin/carddisp.pl?gene=RAB5A) | [3:19947079-19985175](http://mar2017.archive.ensembl.org/Homo_sapiens/Location/View?r=3:19947079-19985175:1) |
| [*DNAL4*](http://genecards.org/cgi-bin/carddisp.pl?gene=DNAL4) | [22:38778508-38794198](http://mar2017.archive.ensembl.org/Homo_sapiens/Location/View?r=22:38778508-38794198:-1) |

Chr, Chromosome

* Gene locating in the X chromosome

**^#^** Data were based on GENCODE release 26 (March 2017) mapped to GRCh37/hg19

**Supplementary Table 2. 82 significant SNPs after false discovery rate (FDR) control (*P*≤0.05)**

| **CHR** | **SNP** | | | ***P**** | ***P*_FDR** | **Mapped gene** |
| --- | --- | --- | --- | --- | --- | --- |
|  | **HGVS Name** | **rs ID** | |  |  |  |
| 10 | g.116979803T>C^#^ | | rs1905539 | 8.40E-07 | 3.544E-03 | *SHTN1* |
| 10 | g.116884159G>A^#^ | | rs2257791 | 9.24E-07 | 3.544E-03 | *SHTN1* |
| 10 | g.116936755A>T^#^ | | rs4751614 | 1.52E-06 | 3.635E-03 | *SHTN1* |
| 10 | g.116919201C>A^#^ | | rs4752018 | 1.90E-06 | 3.635E-03 | *SHTN1* |
| 10 | g.116955888G>A^#^ | | rs7902527 | 2.61E-06 | 4.000E-03 | *SHTN1* |
| 10 | g.117077171T>A^#^ | | rs11197887 | 3.76E-06 | 4.811E-03 | *SHTN1* |
| 10 | g.117084676A>G^#^ | | rs11197892 | 4.70E-06 | 5.081E-03 | *SHTN1* |
| 10 | g.117037960A>G^#^ | | rs7902502 | 5.30E-06 | 5.081E-03 | *SHTN1* |
| 10 | g.117041514A>G^#^ | | rs7092957 | 6.45E-06 | 5.498E-03 | *SHTN1* |
| 10 | g.117101949A>T^#^ | | rs75834010 | 1.40E-05 | 6.506E-03 | *SHTN1* |
| 10 | g.117075480C>T^#^ | | rs4752028 | 1.53E-05 | 6.506E-03 | *SHTN1* |
| 10 | g.117051929G>A^#^ | | rs9664776 | 1.57E-05 | 6.506E-03 | *SHTN1* |
| 10 | g.117116690C>T^#^ | | rs61873047 | 2.14E-05 | 6.506E-03 | *SHTN1* |
| 10 | g.117047383T>A^#^  (g.117047383T>C^#^) | | rs2420302 | 2.31E-05 | 6.506E-03 | *SHTN1* |
| 10 | g.117040596A>G^#^ | | rs4752026 | 2.37E-05 | 6.506E-03 | *SHTN1* |
| 10 | g.117071030C>T^#^ | | rs77911460 | 2.75E-05 | 6.506E-03 | *SHTN1* |
| 10 | g.117101266G>T^#^ | | rs17095681 | 2.75E-05 | 6.506 E-03 | *SHTN1* |
| 10 | g.117092495T>C^#^ | | rs17095666 | 3.10E-05 | 6.506E-03 | *SHTN1* |
| 10 | g.117041445C>G^#^ | | rs1898358 | 3.30E-05 | 6.506E-03 | *SHTN1* |
| 10 | g.117103632G>A^#^ | | rs142626538 | 3.36E-05 | 6.506E-03 | *SHTN1* |
| 10 | g.117069316G>A^#^  (g.117069316G>C^#^) | | rs117030104 | 3.75E-05 | 6.506E-03 | *SHTN1* |
| 10 | g.117076543G>A^#^ | | rs718371 | 3.75E-05 | 6.506E-03 | *SHTN1* |
| 10 | g.117076569T>C^#^ | | rs718372 | 3.75E-05 | 6.506E-03 | *SHTN1* |
| 10 | g.117084505G>A^#^  (g.117084505G>C^#^) | | rs191389574 | 3.75E-05 | 6.506E-03 | *SHTN1* |
| 10 | g.117086295T>C^#^ | | rs79319384 | 3.75E-05 | 6.506E-03 | *SHTN1* |
| 10 | g.117086685A>C^#^ | | rs80056111 | 3.75E-05 | 6.506E-03 | *SHTN1* |
| 10 | g.117091941A>C^#^ | | rs17095665 | 3.75E-05 | 6.506E-03 | *SHTN1* |
| 10 | g.117092850C>T^#^ | | rs80089823 | 3.75E-05 | 6.506E-03 | *SHTN1* |
| 10 | g.117094897C>A^#^ | | rs17095673 | 3.75E-05 | 6.506E-03 | *SHTN1* |
| 10 | g.117095719G>A^#^ | | rs17095675 | 3.75E-05 | 6.506E-03 | *SHTN1* |
| 10 | g.117095851C>T^#^ | | rs17095678 | 3.75E-05 | 6.506E-03 | *SHTN1* |
| 10 | g.117099128T>G^#^ | | rs76228171 | 3.75E-05 | 6.506E-03 | *SHTN1* |
| 10 | 117076044G>A^#^ | | rs150876516 | 3.79E-05 | 6.506E-03 | *SHTN1* |
| 10 | g.117068305G>A^#^ | | rs141296137 | 3.96E-05 | 6.506E-03 | *SHTN1* |
| 10 | g.117108857T>C^#^ | | rs76919131 | 4.01E-05 | 6.506E-03 | *SHTN1* |
| 10 | g.117112119C>T^#^ | | rs142516585 | 4.01E-05 | 6.506E-03 | *SHTN1* |
| 10 | g.117113779A>C^#^ | | rs149127348 | 4.01E-05 | 6.506E-03 | *SHTN1* |
| 10 | g.117116046A>G^#^ | | rs12572971 | 4.01E-05 | 6.506E-03 | *SHTN1* |
| 10 | g.117122038T>C^#^ | | rs144351843 | 4.01E-05 | 6.506E-03 | *SHTN1* |
| 10 | g.117039219C>G^#^  (g.117039219C>T^#^) | | rs10787743 | 4.24E-05 | 6.506E-03 | *SHTN1* |
| 10 | g.117043468C>T^#^ | | rs7077210 | 4.24E-05 | 6.506E-03 | *SHTN1* |
| 10 | g.117048665C>T^#^ | | rs1898352 | 4.24E-05 | 6.506E-03 | *SHTN1* |
| 10 | g.117050840A>G^#^ | | rs10886033 | 4.24E-05 | 6.506E-03 | *SHTN1* |
| 10 | g.117051415A>T^#^ | | rs1946761 | 4.24E-05 | 6.506E-03 | *SHTN1* |
| 10 | g.117052881A>G^#^ | | rs9665549 | 4.24E-05 | 6.506E-03 | *SHTN1* |
| 10 | g.117053165A>C^#^ | | rs7922228 | 4.24E-05 | 6.506E-03 | *SHTN1* |
| 10 | g.117054893T>C^#^ | | rs7089319 | 4.24E-05 | 6.506E-03 | *SHTN1* |
| 10 | g.117105961C>T^#^ | | rs744937 | 4.69E-05 | 7.056E-03 | *SHTN1* |
| 10 | g.117084711G>A^#^  (g.117084711G>C^#^) | | rs141293680 | 4.84E-05 | 7.137E-03 | *SHTN1* |
| 10 | g.117118640A>C^#^  (g.117118640A>G^#^)  (g.117118640A>T^#^) | | rs4417186 | 4.99E-05 | 7.200E-03 | *SHTN1* |
| 10 | g.117122525T>C^#^ | | rs77915700 | 5.13E-05 | 7.200E-03 | *SHTN1* |
| 10 | g.117045588G>T^#^ | | rs4123200 | 5.72E-05 | 7.793E-03 | *SHTN1* |
| 10 | g.117036357T>A^#^  (g.117036357T>C^#^) | | rs10749234 | 5.79E-05 | 7.793E-03 | *SHTN1* |
| 10 | g.117042199C>G^#^ | | rs10886030 | 7.69E-05 | 1.011E-02 | *SHTN1* |
| 10 | g.117056026T>G^#^ | | rs4751616 | 7.78E-05 | 1.011E-02 | *SHTN1* |
| 10 | g.117028063A>C^#^ | | rs116894313 | 8.37E-05 | 1.070E-02 | *SHTN1* |
| 10 | g.117103698G>A^#^ | | rs10886042 | 9.01E-05 | 1.132E-02 | *SHTN1* |
| 10 | g.117102909T>C^#^ | | rs10510026 | 9.62E-05 | 1.190E-02 | *SHTN1* |
| 10 | g.117020749G>A^#^ | | rs76916288 | 1.104E-04 | 1.323E-02 | *SHTN1* |
| 10 | g.117020836C>T^#^ | | rs117442234 | 1.104E-04 | 1.323E-02 | *SHTN1* |
| 10 | g.117099349C>A^#^ | | rs76120051 | 1.327E-04 | 1.558E-02 | *SHTN1* |
| 10 | g.117108434A>T^#^ | | rs12219688 | 1.361E-04 | 1.558E-03 | *SHTN1* |
| 10 | g.117109430G>T^#^ | | rs1904302 | 1.361E-04 | 1.558E-03 | *SHTN1* |
| 10 | g.117110375C>G^#^  (g.117110375C>T^#^) | | rs57787663 | 1.579E-04 | 1.780E-02 | *SHTN1* |
| 10 | g.117068049G>A^#^ | | rs7078160 | 1.641E-04 | 1.780E-02 | *SHTN1* |
| 10 | g.117086783C>G^#^ | | rs10886040 | 1.654E-04 | 1.780E-02 | *SHTN1* |
| 10 | g.117071699A>G^#^  (g.117071699A>T^#^) | | rs10886037 | 1.671E-04 | 1.780E-02 | *SHTN1* |
| 10 | g.117034380G>A^#^ | | rs11197870 | 1.826E-04 | 1.918E-02 | *SHTN1* |
| 10 | g.117059758A>G^#^ | | rs10886036 | 1.913E-04 | 1.975E-02 | *SHTN1* |
| 10 | g.117051905T>C^#^ | | rs61873019 | 1.932E-04 | 1.975E-02 | *SHTN1* |
| 10 | g.117102213C>A^#^  (g.117102213C>T^#^) | | rs11197899 | 2.099E-04 | 2.119E-02 | *SHTN1* |
| 10 | g.117043205C>T^#^ | | rs7076770 | 2.266E-04 | 2.257E-02 | *SHTN1* |
| 10 | g.117057556C>T^#^ | | rs1898349 | 2.335E-04 | 2.296E-02 | *SHTN1* |
| 17 | g.35720163G>C^§^ | | rs75656820 | 2.404E-04 | 2.334E-02 | *AP2B1* |
| 10 | g.117054366C>T^#^ | | rs9787575 | 2.463E-04 | 2.361E-02 | *SHTN1* |
| 10 | g.116889932T>C^#^ | | rs61872990 | 2.570E-04 | 2.434E-02 | *SHTN1* |
| 10 | g.117066930C>T^#^ | | rs10787751 | 2.839E-04 | 2.656E-02 | *SHTN1* |
| 10 | g.117031015G>A^#^  (g.117031015G>C^#^) | | rs142027484 | 2.990E-04 | 2.763E-02 | *SHTN1* |
| 10 | g.116999591G>A^#^ | | rs11197864 | 3.271E-04 | 2.952E-02 | *SHTN1* |
| 10 | g.117047240T>C^#^ | | rs11197875 | 3.470E-04 | 3.095E-02 | *SHTN1* |
| 10 | g.116881885G>T^#^ | | rs11197843 | 4.450E-04 | 3.923E-02 | *SHTN1* |
| 1 | g.156864512G>A^¢^ | | rs1800877 | 5.534E-04 | 4.823E-02 | *NTRK1* |

CHR, Chromosome

*P*_FDR, *P* value after the false discovery rate (FDR) control

^*^ Primary *P* value before multiple testing correction

^#^ NC_000010.11 (Homo sapiens chromosome 10, GRCh38.p12)

^§^ NC_000017.11 (Homo sapiens chromosome 17, GRCh38.p12)

^¢^ NC_000001.11 (Homo sapiens chromosome 1, GRCh38.p12)

**Supplementary Table 3. Association of SNPs in moderate to high LD (r^2^≥0.5) with the lead SNP at the 10q25.3, 17q12 and 1q23.1**

| **Lead SNP** | | **Chr Loci** | | **SNPs with r^2^≥0.5** | | | **LD (r^2^)** | | **OR(95% CI)** | ***P^*^*** | ***P*^#^** | |  |
| --- | --- | --- | --- | --- | --- | --- | --- | --- | --- | --- | --- | --- | --- |
| **HGVS Name** | **rs ID** |  |  | **HGVS Name** | **rs ID** | |  |  |  |  |  |  |  |
| g.116979803T>C^￡^ | rs1905539 | 10q25.3 | | g.116979803T>C^￡^ | | rs1905539 | - | 1.652 (1.353-2.018) | | 8.40E-07 | | *-* | |
|  |  | |  | g.116884159G>A^￡^ | | rs2257791 | 0.923169 | 1.624(1.338-1.972) | | 9.24E-07 | | 0.7814 | |
|  |  |  |  | g.116936755A>T^￡^ | | rs4751614 | 0.923029 | 1.623(1.332-1.977) | | 1.52E-06 | | 0.6218 | |
|  |  |  |  | g.116919201C>A^￡^ | | rs4752018 | 0.949466 | 1.613(1.325-1.963) | | 1.90E-06 | | 0.4096 | |
|  |  |  |  | g.116955888G>A^￡^ | | rs7902527 | 0.961872 | 1.619(1.324-1.979) | | 2.61E-06 | | 0.3687 | |
|  |  |  |  | g.117077171T>A^￡^ | | rs11197887 | 0.514801 | 1.565(1.294-1.892) | | 3.76E-06 | | 0.2162 | |
|  |  |  |  | g.117084676A>G^￡^ | | rs11197892 | 0.524367 | 1.558(1.288-1.883) | | 4.70E-06 | | 0.3055 | |
|  |  |  |  | g.117041514A>G^￡^ | | rs7092957 | 0.515449 | 1.547(1.280-1.870) | | 6.45E-06 | | 0.3248 | |
|  | | |  |  | |  |  |  | |  | |  | |
| g.117037960A>G^￡^ | rs7902502 | | 10q25.3 | g.117037960A>G^￡^ | | rs7902502 | - | 0.5125(0.3843-0.6834) | | 5.30E-06 | | - | |
|  |  |  |  | g.117051929G>A^￡^ | | rs9664776 | 0.903184 | 0.5280(0.3951-0.7055) | | 1.57E-05 | | 0.9277 | |
|  |  |  |  | g.117047383T>A^￡^  (g.117047383T>C^￡^) | | rs2420302 | 0.80953 | 0.5267(0.3914-0.7087) | | 2.31E-05 | | 0.3353 | |
|  |  |  |  | g.117040596A>G^￡^ | | rs4752026 | 0.923799 | 0.5351(0.4004-0.7151) | | 2.37E-05 | | 0.6242 | |
|  |  |  |  | g.117041445C>G^￡^ | | rs1898358 | 0.923799 | 0.5361(0.3994-0.7195) | | 3.30E-05 | | 0.1956 | |
|  |  |  |  | g.117039219C>G^￡^  (g.117039219C>T^￡^) | | rs10787743 | 0.923799 | 0.5455(0.4082-0.7292) | | 4.24E-05 | | 0.2426 | |
|  |  |  |  | g.117043468C>T^￡^ | | rs7077210 | 0.923799 | 0.5455(0.4082-0.7292) | | 4.24E-05 | | 0.2426 | |
|  |  |  |  | g.117048665C>T^￡^ | | rs1898352 | 0.923799 | 0.5455(0.4082-0.7292) | | 4.24E-05 | | 0.2426 | |
|  |  |  |  | g.117050840A>G^￡^ | | rs10886033 | 0.961383 | 0.5455(0.4082-0.7292) | | 4.24E-05 | | 0.2426 | |
|  |  |  |  | g.117051415A>T^￡^ | | rs1946761 | 0.923799 | 0.5455(0.4082-0.7292) | | 4.24E-05 | | 0.2426 | |
|  |  |  |  | g.117052881A>G^￡^ | | rs9665549 | 0.961383 | 0.5455(0.4082-0.7292) | | 4.24E-05 | | 0.2426 | |
|  |  |  |  | g.117053165A>C^￡^ | | rs7922228 | 0.923799 | 0.5455(0.4082-0.7292) | | 4.24E-05 | | 0.2426 | |
|  |  |  |  | g.117054893T>C^￡^ | | rs7089319 | 0.923799 | 0.5455(0.4082-0.7292) | | 4.24E-05 | | 0.2426 | |
|  |  |  |  | g.117118640A>C^￡^  (g.117118640A>G^￡^)  (g.117118640A>T^￡^) | | rs4417186 | 0.903759 | 0.5455(0.4011-0.7274) | | 4.99E-05 | | 0.4435 | |
|  |  |  |  | g.117045588G>T^￡^ | | rs4123200 | 0.961383 | 0.5455(0.4135-0.7374) | | 5.72E-05 | | 0.176 | |
|  |  |  |  | g.117036357T>A^￡^  (g.117036357T>C^￡^) | | rs10749234 | 0.923799 | 0.5500(0.411-0.7361) | | 5.79E-05 | | 0.2403 | |
|  |  |  |  | g.117042199C>G^￡^ | | rs10886030 | 0.923799 | 0.5589(0.4189-0.7457) | | 7.69E-05 | | 0.1279 | |
|  |  |  |  | g.117056026T>G^￡^ | | rs4751616 | 0.923799 | 0.5602(0.4203-0.7468) | | 7.78E-05 | | 0.2164 | |
|  |  |  |  | g.117028063A>C^￡^ | | rs116894313 | 0.742969 | 0.5254(0.3813-0.724 | | 8.37E-05 | | 0.8389 | |
|  |  |  |  | g.117020749G>A^￡^ | | rs76916288 | 0.724959 | 0.5309(0.3851-0.7318) | | 1.104E-04 | | 0.8623 | |
|  |  |  |  | g.117020836C>T^￡^ | | rs117442234 | 0.724959 | 0.5309(0.3851-0.7318) | | 1.104E-04 | | 0.8623 | |
|  |  |  |  | g.117099349C>A^￡^ | | rs76120051 | 0.676022 | 0.5155(0.3670-0.7242) | | 1.327E-04 | | 0.4104 | |
|  |  |  |  | g.117031015G>A^￡^  (g.117031015G>C^￡^) | | rs142027484 | 0.675044 | 0.5053(0.3490-0.7315) | | 2.99 E-04 | | 0.8412 | |
|  | | |  |  | |  |  |  | |  | |  | |
| g.35720163G>C^§^ | rs75656820 | | 17q12 | g.35720163G>C^§^ | | rs75656820 | - | 0.4574(0.3013-0.6944) | | 2.40E-04 | | *-* | |
|  | | |  |  | |  |  |  | |  | |  | |
| g.156864512G>A^¢^ | rs1800877 | | 1q23.1 | g.156864512G>A^¢^ | | rs1800877 | - | 1.969(1.340-2.892) | | 5.53E-04 | | *-* | |

LD was based on 1000Genome Project November 2014 release CHB+JPT population information

Chr, Chromosome

OR, odds ratio

CI, Confidence interval

*^*^* Primary *P* value

^#^ *P* value for conditional analysis on the lead SNP

^￡^ NC_000010.11 (Homo sapiens chromosome 10, GRCh38.p12)

^§^ NC_000017.11 (Homo sapiens chromosome 17, GRCh38.p12)

^¢^ NC_000001.11 (Homo sapiens chromosome 1, GRCh38.p12)

**Supplementary Table 4. Function annotation and prediction of four lead SNPs**

| **Lead SNP** | | | **Chr Position** | **Regulome Score** | **Mapped Gene** | **DbSNP function annotation** | **RegPotential^$^** |
| --- | --- | --- | --- | --- | --- | --- | --- |
| **HGVS Name** | **rs ID** | |
| g.116979803T>C^￡^ | | rs1905539 | Chr10: 18739064-118739564 | 5^#^ | *SHTN1* | intronic | - |
| g.117037960A>G^￡^ | | rs7902502 | Chr10: 118797221-118797721 | 6^#^ | *SHTN1* | intronic | - |
| g.35720163G>C^§^ | | rs75656820 | Chr17: 34046932-34047432 | 5^#^ | *AP2B1* | intronic | - |
| g.156864512G>A^¢^ | | rs1800877 | Chr1: 156834054-156834554 | 5^#^ | *NTRK1* | intronic | 0.269336 |

Chr, chromosome

^$^ Regulatory Potential Scores

^#^ Minimal transcription factor binding evidence

^￡^ NC_000010.11 (Homo sapiens chromosome 10, GRCh38.p12)

^§^ NC_000017.11 (Homo sapiens chromosome 17, GRCh38.p12)

^¢^ NC_000001.11 (Homo sapiens chromosome 1, GRCh38.p12)
